# Incentive motivation in first-episode psychosis: A behavioural study

**DOI:** 10.1186/1471-244X-8-34

**Published:** 2008-05-08

**Authors:** Graham K Murray, Luke Clark, Philip R Corlett, Andrew D Blackwell, Roshan Cools, Peter B Jones, Trevor W Robbins, Luise Poustka

**Affiliations:** 1 Brain Mapping Unit, Department of Psychiatry, University of Cambridge, Cambridge, UK; 2 CAMEO, Cambridgeshire and Peterborough Mental Health Partnership NHS Trust, Cambridge, UK; 3 Behavioural and Clinical Neuroscience Institute, University of Cambridge, Cambridge, UK; 4 Cambridge Cognition Limited, Cambridge, UK; 5 Klinik für Psychiatrie und Psychotherapie des Kindes- und Jugendalters, Zentralinstitut für Seelische Gesundheit, Mannheim, Germany

## Abstract

**Background:**

It has been proposed that there are abnormalities in incentive motivational processing in psychosis, possibly secondary to subcortical dopamine abnormalities, but few empirical studies have addressed this issue.

**Methods:**

We studied incentive motivation in 18 first-episode psychosis patients from the Cambridge early psychosis service CAMEO and 19 control participants using the Cued Reinforcement Reaction Time Task, which measures motivationally driven behaviour. We also gathered information on participants' attentional, executive and spatial working memory function in order to determine whether any incentive motivation deficits were secondary to generalised cognitive impairment.

**Results:**

We demonstrated the anticipated "reinforcement-related speeding" effect in controls (17 out of 19 control participants responded faster during an "odd-one-out" task in response to a cue that indicated a high likelihood of a large points reward). Only 4 out of 18 patients showed this effect and there was a significant interaction effect between reinforcement probability and diagnosis on reaction time (F_1,35 _= 14.2, p = 0.001). This deficit was present in spite of preserved executive and attentional function in patients, and persisted even in antipsychotic medication free patients.

**Conclusion:**

There are incentive motivation processing abnormalities in first-episode psychosis; these may be secondary to dopamine dysfunction and are not attributable to generalised cognitive impairment.

## Background

Motivational problems such as avolition have been noted in schizophrenia since the initial descriptions of the illness [1]. Proposed neurological models of psychosis have linked schizophrenic motivational deficits to hypofrontality, but an alternative hypothesis is that motivational dysfunction in schizophrenia and other psychoses is underpinned by abnormal activity of subcortical monoamine systems [2-4]. In particular, ascending midbrain dopamine neurons are known to play a key role in incentive motivation [5,6] and to signal unexpected reward and errors in reward prediction [7]. Reward system dysfunction may underlie not only avolition but also, or alternatively, other psychotic symptoms including stereotyped patterns of thought and behaviour [8] and delusional beliefs [8-13]. For example, it has been argued that dysregulated midbrain dopamine neuron firing could result in an individual maladaptively attributing motivational importance to innocuous stimuli or events, i.e. experiencing abnormal referential ideas [10,11]. An affected individual, having experienced abnormally salient phenomena secondary to dysregulated dopamine, may then impose a "top-down" cognitive explanation onto such experiences in order to make sense of them, potentially culminating in a delusion [10]. Alternatively, dysregulated dopamine neuron firing could result in an amplification of the salience of an internally generated voice, which could in turn lead to an abnormal perception [14].

In spite of such speculations that disrupted reward and motivational processing may underpin positive and/or negative psychotic symptoms, it has yet to be clearly demonstrated whether such disruptions are present in psychosis or not, let alone whether such disruptions relate to symptom expression. A major challenge in evaluating the hypothesis that reward processing is abnormal in psychosis is the lack of available behavioural measures to assess reward processing and incentive motivational processes in humans; the consequence has been that, to date, such processes have only been addressed indirectly in behavioural studies. Patients with schizophrenia display a range of abnormalities of classic associative learning phenomena including Kamin blocking and latent inhibition [15,16], and these abnormalities are responsive to short-term antipsychotic treatment, consistent with a dopaminergic mechanism. In addition, reward-based decision-making on the Iowa Gambling Test (IGT) has been shown to be impaired in psychosis [17] and these effects are also sensitive to medication status [18], although there have been some failures to replicate the case-control difference [19], and the IGT requires several cognitive processes in addition to reward sensitivity.

In the present study, we sought to examine incentive motivation in psychosis, using a simple choice reaction time task (the Cued Reinforcement Reaction Time Task, or CRRT [20]) in which healthy participants have previously shown speeding of responses after presentation of coloured cues signalling higher probability of reward: 'reinforcement-related speeding'. Thus a particular cue (an initially and objectively neutral piece of information) becomes associated with enhanced likelihood of reinforcement, and so stimulates more effortful (i.e. rapid) responding in an adaptive performance of the task. We administered the CRRT to a group of patients with first-episode psychosis, in addition to a number of neuropsychological tests of attentional and executive function. By studying first-episode cases, we were able to explore motivational processes in the absence of substantial global cognitive impairment. We hypothesised that patients with psychosis would be less sensitive than healthy participants to the motivational manipulation, and would therefore show attenuated or absent 'reinforcement-related speeding' on the task.

## Methods

### Participants

18 individuals (mean age 23; 9 men) with first-episode psychosis were recruited from the Cambridge first episode psychosis service, CAMEO for the study. Inclusion criteria for CAMEO is age between 17 and 35, suffering from a first episode of psychosis as defined by the Melbourne criteria of the presence of psychotic symptoms for at least one week [21], and duration of antipsychotic treatment of under 6 months at the time of initial assessment. Nineteen healthy volunteers (mean age 25; 9 men) were recruited from the general population by advertisement to act as a control group. Eleven of the 18 patients were taking antipsychotic medication; all of these 11 were taking "atypical" antipsychotic agents with a mean chlorpromazine equivalent dose of 264 mg. Of these, 3 were taking olanzapine (10 mg daily), 2 risperidone (1 mg daily and 3 mg daily), 2 quetiapine (500 mg daily and 400 mg daily), 1 clozapine (400 mg daily), 2 aripiprazole (10 mg daily and 15 mg daily), and 1 amisulpride (200 mg daily). Of the 7 antipsychotic-free patients, 5 were taking no medication, 1 was taking sertraline and 1 sodium valproate. Only 1 of the antipsychotic free patients had previously briefly taken antipsychotics, but was antipsychotic free for 2 weeks prior to assessment. After referral to the service, we waited until clinical presentation at least partially stabilised before commencing the study (over 75% studied within 5 months of referral, all assessed within a year of referral). As a consequence most patients in this study had mild symptoms at the time of the experiment: mean Brief Psychiatric Rating Scale (BPRS) positive symptom score 1.8 (very mild) and mean BPRS negative symptom score 1.8 (very mild). BPRS scores were unavailable on two patients. Twelve months after the experiment, a psychiatrist (GM) assigned DSM-IV diagnoses to patients using all available clinical information; 9 patients met criteria for schizophrenia, 2 for schizoaffective disorder, 5 for bipolar disorder, 1 for delusional disorder and 1 for psychosis not otherwise specified. This range of diagnoses is broadly representative of outcomes in first episode psychosis services [22]. The research was approved by the local Research Ethics Committee; all participants provided informed consent.

### The Cued Reinforcement Reaction Time Task [20]

**Figure 1:**
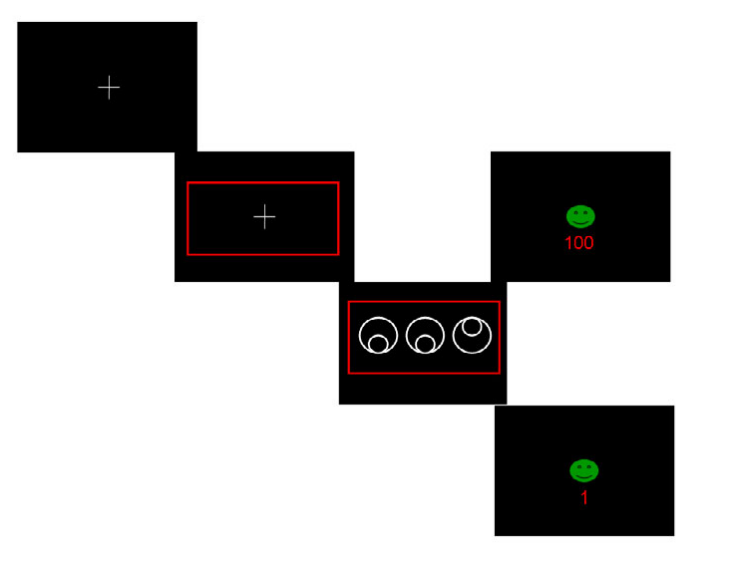
**The CRRT (Cued Reinforcement Reaction Time Task)**. Participants were asked to identify the 'odd-one-out' on each trial as fast as possible without making mistakes. A coloured stimulus window acted as a cue, indicating the probability of receiving reinforcement. Reinforcement was 100 points and a smiley face for a response faster than the cutoff score (top right), 1 point and a smiley face for a response slower than the cutoff score (bottom right), and 0 points and a sad face for an incorrect response (not shown). Reinforcement probabilities were 10, 50, and 90% (depending on the colour of the cue). No feedback was presented and no points were obtained on the remaining (unreinforced) trials.

On each trial, participants perform a rapid 'odd-one-out' judgment on three shapes, one of which is distinct (Figure 1). Stimuli presentation was preceded by a cue (a coloured rectangle of one of 3 colors) that signalled the likelihood that a correct response would be followed by a reward (the three colors were associated with 10, 50, and 90% reinforcement probability). 96 trials were administered in the test, with 32 trials of each cue-type. Responses were made with the dominant hand, and were immediately followed by feedback: correct identification of the odd-one-out yielded a green smiley face; incorrect responses yielded a red sad face. The magnitude of reinforcement was dependent on accuracy and reaction time (RT): a fast correct response was rewarded with 100 points, a slow correct response with 1 point, and an incorrect response with 0 points. Participants were told that they had to obtain as many points as possible and were subsequently given a debrief questionnaire in order to provide a measure of explicit awareness of stimulus-reinforcement contingencies. RT thresholds were titrated for each individual participant in a practice session, which also served to familiarise the participants with the task. In the practice session, which consisted of two blocks of 20 trials, no cues were presented and no trial by trial feedback was provided. The mean RT and standard deviation for the second practice block was used to compute a cut-off for reward delivery in the main task, so that reward attainment levels were titrated to individual subjects' psychomotor speed. The cut-off was calculated by subtracting the standard deviation from the mean RT.

### Additional Neuropsychological Measures

Three further tests (described in Additional file 1: information on additional neuropsychological measures) from the Cambridge Neuropsychological Test Automated Battery (CANTAB) were administered to assess attentional and executive function: the Intra-Dimensional/Extra-Dimensional (ID/ED) Shift test [derived from the Wisconsin Card Sort Test, see 23], the Rapid Visual Information Processing (RVIP) test [derived from the Continuous Performance Test, see 24], and the Spatial Working Memory (SWM) test [25].

### Data Analysis

Demographic characteristics of the two groups were compared using independent-samples t-tests and chi-squared tests. Incorrect trials were excluded from the CRRT reaction time analysis. CRRT performance was assessed using mixed-model ANOVA with group (patients, controls) as a between-subjects factor, and probability of reinforcement (10%, 50%, 90%) as a within-subjects factor. A linear contrast was used to test for reaction time trend across the reinforcement contingencies [26,27]. Debriefing data were analysed with chi-squared tests. In order to attempt to rule the possibility that differences between groups were attributable to the effects of dopamine antagonist medication, we repeated the mixed-model ANOVA having excluded the participants who were taking antipsychotic drugs. The proportion of participants in each group who showed reinforcement-related speeding (responding faster on trials with a high probability of reinforcement than on trials with a low probability of reinforcement) was compared using a Chi-Squared Test when all patients were included and Fisher's exact test when antipsychotic treated patients were excluded.

Skewed data were transformed where possible to enable the use of parametric tests. A logarithmic transformation was used for ID/ED extra-dimensional shift errors and total errors, and for RVIP latency data. A square root transformation was used for SWM between errors and within errors. Fisher's Exact Test was used to compare the number of participants from each group who completed the ID/ED test. The Mann Whitney U-Test was used to compare RVIP response bias data groups. All tests were two-tailed with alpha set at 0.05.

## Results

### Cued Reinforcement Reaction Time Task

**Figure 2:**
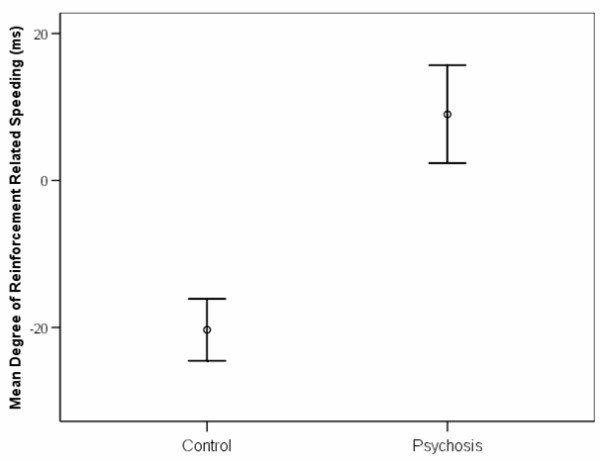
**The degree of reinforcement-related speeding in patients and controls**. The mean degree of reinforcement-related speeding (as measured by the mean reaction time on 90% probability trials minus the mean reaction time on 10% probability trials) differs in patients and controls (t = 3.8, p = 0.001). Error bars represent standard errors of the mean.

**Figure 3:**
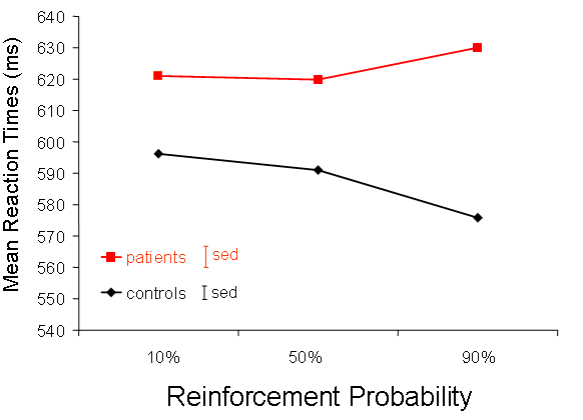
**Reaction time stratified by reinforcement probability in the Cued Reinforcement Reaction Time Task**. This plot demonstrates reinforcement-related speeding in controls (as defined by a linear trend of decreasing reaction time with increasing probability of reinforcement) and an interaction between reinforcement-related speeding and diagnostic group (p = 0.001). Error bars represent standard errors of the mean difference in reaction times on the 90% probability trials compared with the 10% probability trials.

Whilst 17 of the 19 controls showed reinforcement-related speeding (faster reaction times on the high probability trials compared to the low probability trials), only 4 of the 18 patients showed that effect (X^2 ^= 17, p = 0.00004). The *degree *of the reinforcement-related speeding effect is shown in Figures 2 and 3. Analysis of variance revealed no main effect of reinforcement probability (F_2,70 _= 0.6 p = 0.6) or diagnostic group (F_1,35 _= 0.9, p = 0.3), but a significant diagnostic group by reinforcement probability interaction (F_2,70 _= 4.5, p = 0.025). The trend of reaction time across reinforcement contingencies differed in controls compared with patients (F_1,35 _= 14.2, p = 0.001). Control participants displayed a significant effect of reinforcement probability on reaction in a repeated-measures ANOVA (F_2,17 _= 12, p = 0.001), whereas there was no such effect in the psychosis patients (F_2,16 _= 0.9, p = 0.4). Analysis of the error data showed no significant differences between groups (t = 1.2, p = 0.3). When we excluded the patients who were taking antipsychotic medication, the proportion of participants showing reinforcement-related speeding still differed significantly across groups (Fisher's exact test p = 0.006) and the effect of differing reaction time trend across reinforcement probabilities in controls and in patients remained significant (F_1,24 _= 4.5, p = 0.045). There was no significant difference between groups in explicit awareness of reinforcement contingencies (p = 0.89): 58% of controls and 66.6% of patients correctly identified the colour most likely to lead to a reward. We also examined whether the mean reaction time from the second block of practice, and the standard deviation of reaction times from the second block of practice differed between groups (as these were used in order to calculate the cut-off thresholds for the task proper). However, there was no difference between groups on practice mean RT (t = 0.3, p = 0.8) or practice RT standard deviation (t = 0.5, p = 0.6).

We then examined the correlation between symptoms and reinforcement-related speeding, but there was no significant relationship (r = 0.3, p = 0.3, negative symptoms; r = 0.03, p = 0.9, positive symptoms).

### Other cognitive test scores (Table 1)

**Table 1:** Cognitive Test Scores

**Test**	**Control**	**Psychosis**	***p***
ID/ED (Proportion completing)*	83%	95%	0.3
ID/ED EDS errors	4.3 (5.9)	7.9 (9.6)	0.2
ID/ED Pre-EDS errors	5.7 (2.2)	6 (2.5)	0.7
ID/ED Total errors	11.3 (6.1)	15.2 (9.8)	0.2
SWM Between Errors	14.3 (13.4)	24.2 (14.4)	0.02
SWM Within Errors	1.1 (1.6)	1.6 (2.1)	0.3
SWM Strategy	26.3 (6.4)	32.3 (6.4)	0.01
RVIP Target Detection	0.94 (0.05)	0.91 (0.05)	0.13
RVIP Response Bias^$^	0.96 (0.04)	0.97 (0.05)	0.3
RVIP Latency	409.7 (80.6)	429.7 (118.5)	0.6

Means (Standard Deviation) are shown for the two groups on the additional cognitive tests, with p-values for statistical tests for group difference t-tests apart from * (Chi-Square) and ^$^(Mann-Whitney U).

there were no significant differences between groups on set-shifting or attentional function. 15 out of 18 patients passed all stages of the ID/ED Test, compared to 18 out of 19 control volunteers (p = 0.3). There was no significant difference between groups on number of extra-dimensional shift errors (t = 1.4, p = 0.2), or on pre-extradimensional shift errors (t = 0.4, p = 0.7) or on total errors (t = 1.4, p = 0.2). In the RVIP, there was no difference between groups in terms of response bias (U = 109, p = 0.2), target detection (t = 1.6, p = 0.13) or latency (t = 0.5, p = 0.6). Patients showed impairment in spatial working memory on strategy score (t = 2.7, p = 0.01) and on between-stage errors (t = 2.5, p = 0.02). Spatial working memory scores were unavailable for 4 control participants due to technical problems. There was no difference between groups on the subsidiary measure of within stage-errors (t = 1, p = 0.3).

We then examined the relationship between incentive motivation and spatial working memory. We performed a logistic regression analysis in patients to test whether the presence of reinforcement-related speeding can be predicted by spatial working memory performance. Neither spatial working memory strategy score (p = 0.3) nor between search error score (p = 0.2) predicted the presence of reinforcement-related speeding. We further examined whether the degree of reinforcement-related speeding (mean reaction time on 90% probability of reinforcement trials – mean reaction time of 10% probability trials, and mean RT on 90% probability trials – mean RT on 50% probability trials) correlated with spatial working memory performance in patients. These correlations were not significant: r = 0.35, p = 0.2 (mean RT 90-50 vs strategy); r = 0.02, p = 0.9 (mean RT 90-10 v strategy); r = 0.1, p = 0.7 (mean RT 90-50 vs between search errors); r = 0.3, p = 0.2 (mean RT 90-10 vs between search errors).

Finally we tested whether patients who ultimately were diagnosed with schizophrenia performed differently on cognitive tests from those ultimately diagnosed with bipolar disorder, but there was no significant difference on any test.

## Discussion

Control participants demonstrated an adaptive behavioural response to cues of varying degree of motivational salience (acquired through association with reward). Whilst the majority of patients were able to report correctly the cue most associated with reward, they did not show the adaptive behavioural reinforcement-related speeding effect of controls. As such, there was a disconnection between their awareness of the environment, and their ability to modulate their behaviour in accordance with that knowledge. This supports the theory that patients with early psychosis show deficits in incentive motivation.

Whilst we follow other authors in arguing that incentive motivation plays a key role in response speeds and latencies during reinforcement tasks in general [28,29] and the CRRT in particular [20,30], we do acknowledge that other cognitive processes also contribute to the CRRT, including attentional and learning processes. We note that these first-episode psychosis patients did form a fairly cognitive intact group, given their good performance on attentional set shifting and rapid information processing. The use of these comparison cognitive assessments shows that the patients' abnormal performance in the CRRT was not purely secondary to generalised cognitive deficits and is likely to truly reflect abnormalities in motivational processing. Patients did show impaired spatial working memory, in accordance with previous evidence documenting spatial working deficits early in the course of psychotic illness [31]. However, patients' spatial working memory deficits did not relate to their performance on the CRRT, indicating that the incentive motivation abnormalities we observed were not confounded by the patients' cognitive deficits. We note that there was a moderate, but non-significant, correlation between spatial working memory strategy and performance on the CRRT. A recent study [32] that investigated motivation processing in chronic medicated schizophrenia also showed a moderate correlation between motivated responding and working memory. It is possible that if we had used a larger sample size we might have seen a significant relationship between CRRT performance and working memory, and this area warrants further study in larger samples.

Some limitations should be noted. The sample size is small, and some of the patients were taking atypical antipsychotic medication, which may have affected the results. However there is evidence that atypical antipsychotic agents do not impair motivational processing in patients with psychosis, but rather such medications may ameliorate underlying abnormalities in reward expectation in the ventral striatum [33]. Furthermore, when we excluded patients who were taking antipsychotic medication from the analysis, a statistically significant difference between groups in incentive motivation remained, which suggests that the abnormality in patients is not solely attributable to dopamine antagonist effects of treatment.

A variety of evidence from studies in both humans and experimental animals indicates that subcortical dopamine systems play a critical role in reward and motivational processing [34,35]. Dopamine may be more critical in motivation, anticipation of rewards and prediction error signalling than in consummatory processing, which has been linked to opioid receptor activation [7,36]. Despite extensive previous theorising attempting to link dopamine dysfunction, abnormalities in reward processing, and psychosis [8,10,14], few experimental behavioural or physiological studies have investigated such theories in patients. In a recent functional MRI study of reward learning, Murray et al [37] showed that brain responses correlating with reward prediction error in the dopaminergic midbrain and associated dopamine neuron striatal and limbic target regions were abnormal in patients with active psychotic symptoms. Juckel and colleagues [38] demonstrated that expectation of reward, when compared with expectation of neutral feedback, is associated with reduced ventral striatal activity in schizophrenia when compared to controls. Taken together, these studies provide preliminary evidence for physiological abnormalities in psychotic illness in learning about and anticipating rewards, combined with an impaired ability to modulate behaviour in response to incentives. We suggest that this impairment may be secondary to dopamine dysfunction, though we acknowledge that, as yet, no direct evidence has proved that performance on the CRRT is dopamine dependent. In contrast to demonstrated anticipatory and motivational deficits, consummatory reward processing in psychosis may be intact [32,39,40].

## Conclusion

This study reports deficits in incentive motivation processing in first episode psychosis. Future studies should examine whether incentive motivation deficits in psychosis are sensitive to pharmacological, especially dopaminergic, modulation.

## Competing interests

AB is an employee of Cambridge Cognition, which supplies the CANTAB cognitive test battery; LC and TWR have acted as consultants for Cambridge Cognition. GKM, PRC, RC, PBJ, and LP declare there are no other competing interests.

## Authors' contributions

GKM, ADB and TWR had the idea for the study. GKM, LP, LC, PRC and PJB designed the study. ADB and RC designed and programmed the CRRT test. LP acquired the data. GM and LP analysed the data. All authors contributed to the drafting and revising of the manuscript.

## Pre-publication history

The pre-publication history for this paper can be accessed here:



## Supplementary Material

Additional file 1 Additional Material consists of "Additional file 1: information on additional neuropsychological measures." Click here for file
