# Supplementary material for: Incentive motivation in first-episode psychosis: A behavioural study
Source: BMC Psychiatry. 2008 May 8;8:34. doi: 10.1186/1471-244X-8-34 (PMC2397419; doi:10.1186/1471-244X-8-34)
Supplement: Additional file 1 — Additional Material consists of "Additional file 1: information on additional neuropsychological measures." [file 1471-244X-8-34-S1.doc]

**Incentive Motivation In First Episode Psychosis:**

**a Behavioural Study**

**Additional file 1: information on additional neuropsychological measures and retrospective power calculation**

Murray GK, Clark L, Corlett PR, Blackwell AD, Cools R, Jones PB, Robbins TW, Poustka L

**Additional Neuropsychological Measures:**

The Intra-Extra Dimensional Set Shift (ID/ED) is an executive function test of rule acquisition and reversal. It features visual discrimination and attentional set formation and tests the maintenance, shifting and flexibility of attention. Two artificial dimensions are used in the test: colour-filled shapes and white lines. Simple stimuli are made up of just one of these dimensions, whereas compound stimuli are made up of both, namely white lines overlying colour-filled shapes. The subject starts by seeing two simple colour-filled shapes, and must learn which one is correct by touching it. Feedback teaches the subject which stimulus is correct, and after six correct responses, the stimuli and/or rules are changed. These shifts are initially intra-dimensional (e.g. colour filled shapes remain the only relevant dimension), then later extra-dimensional (white lines become the only relevant dimension). Subjects progress through the test by satisfying a set criterion of learning at each stage (6 consecutive correct responses). If at any stage the subject fails to reach this criterion after 50 trials, the test terminates. The dependent variables are stages passed, extra-dimensional shift errors and pre-extra-dimensional shift errors.

The Rapid Visual Information Processing Test (RVIP) is RVP is a test of visual sustained attention and is a version of a continuous performance test. A white box appears in the centre of the computer screen, inside which digits, from 2 to 9, appear in a pseudo-random order, at the rate of 100 digits per minute. Subjects are requested to detect target sequences of digits (for example, 2-4-6, 3-5-7, 4-6-8) and to register responses using the press pad. The outcome measures are latency on correct trials, target detection (A’) and response bias (B’’).

The Spatial Working Memory Test (SWM) is a test of the ability to retain spatial information and to manipulate remembered items in working memory. It is a self-ordered task, which also assesses heuristic strategy. The test begins with a number of coloured squares (boxes) being shown on the screen. By touching the boxes and using a process of elimination, the subject should find one blue ‘token’ in each of a number of boxes and use them to fill up an empty column on the right hand side of the screen. The number of boxes is gradually increased, until it is necessary to search a total of eight boxes. The colour and position of the boxes used are changed from trial to trial to discourage the use of stereotyped search strategies. The outcome measures for SWM include errors (between search errors and within search errors) and strategy score. A ‘between-search’ error occurs when a subject returns to a box in which a token has already been found during an earlier search sequence. A ‘within-search’ error occurs when a participant returns to inspect a box already shown to be empty earlier in the current search sequence.

Retrospective Power Calculation:

In order to determine the proportion of patients showing reinforcement related speeding in a Chi-square test, given that 90% of controls show this effect, there is 80% power to detect the alternative hypothesis, with a Type 1 error probability of 0.05, that 50% of patients or less show reinforcement related speeding. As the data show, in fact only 22% of patients showed reinforcement related speeding, thus our sample of 18 patients provided over 95% power.

Dupont WD, Plummer WD: "Power and Sample Size Calculations: A Review and Computer Program", Controlled Clinical Trials 1990; 11:116-28.
